# Inclusion of double helix structural oligonucleotide (STexS) results in an enhance of SNP specificity in PCR

**DOI:** 10.1038/s41598-021-98610-8

**Published:** 2021-09-27

**Authors:** Jae Jong Kim, Hyoung-Min Park, A. Young Kyoung, In Kyung Park, Si-Kyu Lim, Byoung Chul Park

**Affiliations:** 1 GenoTech Corporation, 26-69, Gajeongbuk-ro, Yuseong-gu, Daejeon, 34113 Republic of Korea; 2 Certified Reference Material Lab, Korea Research Institute of Standards and Science, 267 Gajeong-ro, Yuseong-gu, Daejeon, 43113 Republic of Korea; 3 Disease Target Structure Research Center, Korea Research Institute of Bioscience and Biotechnology, Daejeon, 34141 Republic of Korea

**Keywords:** Biological techniques, Cancer, Molecular biology

## Abstract

Genetic mutations such as single nucleotide polymorphisms (SNP) are known as one of the most common forms which related to various genetic disorders and cancers. Among of the methods developed for efficient detection of such SNP, polymerase chain reaction (PCR) methods are widely used worldwide for its cost and viable advantages. However, the technique to discriminate small amounts of SNP mixed in abundant normal DNA is incomplete due to intrinsic technical problems of PCR such as amplification occurring even in 3’mismatched cases because of high enzyme activity of DNA polymerases. To overcome the issue, specifically designed PCR platform, STexS (SNP typing with excellent specificity) using double stranded oligonucleotides was implemented as a means to emphasize the amplification of SNP templates by decreasing unwanted amplification of 3’mismatched DNA copies. In this study, the results indicate several EGFR mutations were easily detected specifically utilizing the STexS platform. Further trials show the novel method works effectively to discriminate mutations in not only general allele specific (AS)-PCRs, but also amplification refractory mutation system (ARMS)-PCR. The STexS platform will give aid in PCRs targeting potential SNPs or genetically mutated biomarkers in human clinical samples.

## Introduction

The investigation of genetic variations which involves in genetic disorders and cancers is essential for predicting cures, establishing treatment methods, and observing prognosis and relapse. Identifying these variations are not only critical for human welfare, but also for the selection and breeding of species and origin verification in agriculture^1^.

Genetic variation can be caused by indigenous or environmental factors which living organisms are exposed throughout its lifespan. The most common form occurring in humans is the single nucleotide polymorphism (SNP) variant, which alters a single nucleotide within an individual. While the process of substitution of the nucleotides are constantly occurred among men, specific SNPs located in a critical genomic region tend to inflict differences in the susceptibility of various diseases such as sickle-cell anemia, cystic fibrosis, and Alzheimer’s disease^2–4^. Studies regarding the discrimination of crucial SNPs are constant. The most common, efficient and viable method is utilizing the polymerase chain reaction (PCR). Nowadays the ability to estimate the nucleotide amount by PCR and quantitative real time PCR (qPCR) is widely used in public health, agriculture and environment preservation^5^. Although the advantage is certain, PCR also has shown limitations. The continuous effort to improve the overall mixture was mainly aimed to enhance reactions, diminish unnecessary primer dimers, reduce nonspecific binds due to mismatches, and block efficiency drops occurred by high G/C rates^6,7^. While most of the issues were solved, primer template mismatches constantly tamper the overall specificity, resulting in unwanted false-positives. For example, “HotStart PCR” was implemented to block unwanted nonspecific amplifications caused by primer binding on unwanted target DNA (not alleles) at low temperature^8^, but this method still is not adequate for identifying a handful of mutant DNA mixed in abundant normal wild-type DNA (alleles). As a result, methods for diagnosing cancers and other disorders using tissue, blood, and saliva are yet to be complete due to the fact that clinical samples are mainly consisted of normal DNA alongside a mere fraction of mutated DNA.

The context of discriminating a mutant within a clinical sample has to have high specificities which can detect 1% or less included mutated DNA alongside a high robustness for further clinical implementations. For these reasons, this study represents a refined method of PCR, STexS (SNP typing with excellent specificity) by adding a double helix forming oligonucleotide DNA (discrimination boosting oligonucleotides; “dbOligo”) to increase the discrimination between matched and mismatched PCR (Sup Fig. 1).

## Results

### Utilizing double stranded oligonucleotides in PCRs for discrimination boosting

The typical allele specific PCR (AS-PCR) proceeds even when a mismatch occurs in the 3’ end of a primer. This is due to DNA polymerase reactions carrying on the next dNTP even when a mismatch happens, which after the next cycle converts to a normal match that continues amplifying. To overcome this issue, the annealing has to be repressed when the initial template detects a mismatch. The priority was to control the amount of DNA polymerase for adjusting reaction rates and additional melting temperature (Tm) calibrations above the lowest annealing temperature for discarding unwanted dimers.

**Figure 1:**
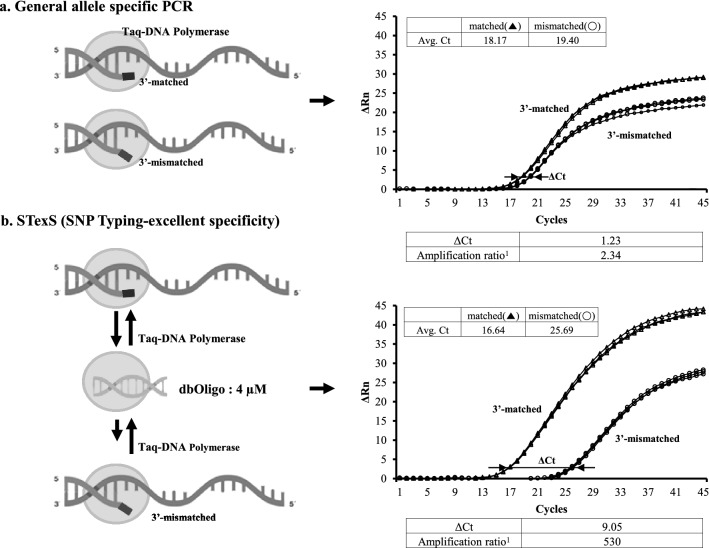
Standard PCR model of DNA polymerase reaction utilizing dbOligo. (**a**) Schematics of general allele specific PCR (AS-PCR). (**b**) Schematics of the SNP Typing -excellent specificity (STexS) PCR. qPCRs were performed with 790-F58-1 for EGFR T790M, and 4 uM of AHP as dbOligo for STexS PCR was used.

To increase the amplification ratio [2^ΔCt^; ΔCt = Ct of 3′ mismatched (normal DNA)—Ct of 3′ matched (mutant DNA)] of qPCR, specifically designed dbOligo, a double helix forming oligonucleotide DNA, was added (Fig. 1). Optimization for the duplex forming ratio required each dbOligo to be designed at least 10 base pairs and adjusted to elevate the melting temperature higher than an average annealing temperature. Further optimization gave evidence in which among various factors such as Tm, length and GC contents, Tm represented to be much more crucial as GC contents and length factors become irrelevant once Tm is above the PCR extension temperature (Sup Figs. 2, 3, 4). In a 3′ matched situation, a general PCR and dbOligo added PCR (STexS-PCR) did not show any big difference. Both Cts were similar (3′ matched in Fig. 1). This implies adding dbOligo within a PCR mixture does not influence the activity of the DNA polymerase. In a 3′ mismatch situation, the K_cat2d_ of dbOligo-added PCR should still be same or slightly lower than the K_cat2_ of a general although highly lower than K_cat1_ and K_cat1d_ in a 3′ match situation PCR (K_cat2_ ≒ K_cat2d_ <  <  < K_cat1_ ≒ K_cat1d_). However, dbOligo-added PCR plot was highly decreased in a in a 3’ mismatch situation (3’ mismatched in Fig. 1 and Sup Fig. 2). The cause is considered to be the decrease of the association reaction (k_2_) or the increase of the dissociation reaction (k_-2_) within the DNAP·P/T_2_ cluster (Sup Fig. 5c, d). In other words, the overall attach and detach rate of DNAP should have been altered by addition of dbOligo (K_-2_/K_2_ <  <  < K_-2d_/K_2d_). Due to these reasons, dbOligo-added PCR easily repress 3′ mismatched amplification without altering 3′ matched PCR.

### qPCR validation of the STexS dbOligo

**Figure 2:**
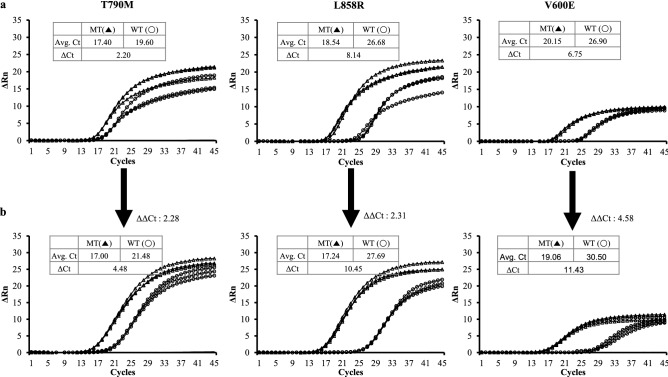
Efficiency test of standard AS-PCR and STexS utilized PCR. qPCR plots of general (**a**) and STexS PCR (**b**). Each Primer was designed to target three types of variation of the gene EGFR; T790M, L858R and BRAF V600E. Each general PCR/STexS PCR was performed to evaluate whether the primer can effectively differentiate matched against mismatched templates. qPCRs were performed with 790-F58-1 for EGFR T790M, 790-F58-1 for EGFR T790M 858-F25 for EGFR L858R and 1799-F11 for BRAF V600E, and 1 uM of AHP3 as dbOligo for STexS PCR was used.

To further address the significance of dbOligo added PCR, the term will be named as SNP Typing-excellent specificity (STexS) PCR. For further validation of STexS, several trials of PCR were performed targeting several variations of the EGFR gene. The forward primers were designed to match or mismatch 1 base pair of the EGFR c.2369 C > T (p.T790M); EGFR c.2573T > G (p.L858R) and BRAF c.1799 rc. A > T (p.V600E) (Sup Table 1). In a general AS-PCR, the discrepancy of wild-type and mutant qPCR versions were not sufficient to address apparent difference. When STexS was implemented, the contrast between matched and mismatched were escalated with increased ΔΔCt values (ΔΔCt = ΔCt of not STEX PCR—ΔCt of STEX PCR) between 2.20 to a maximum of 4.48 (Fig. 2). Repeated trials of each method consistently represented STexS PCR repressing non-significant templates (Sup Fig. 6). The increased discrimination of EGFR T790M and wild-type was enough to doubtlessly consider the matched template of the mutated variate was differentially sorted with STexS PCR.

**Figure 3:**
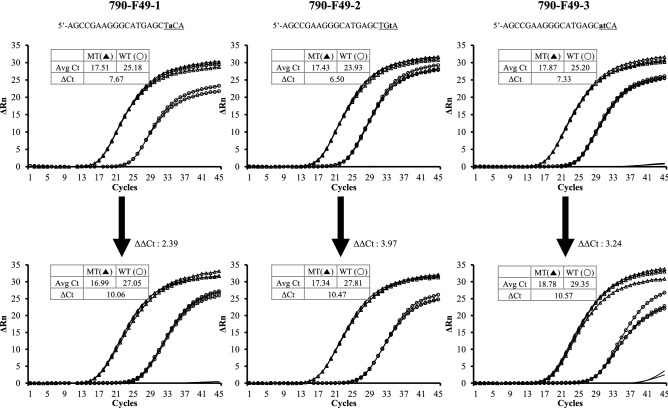
Efficiency of ARMS-PCR using standard and STexS utilized methods. Each ARMS primer is designed to target the EGFR gene variation T790M. Additional one or two mismatch base(s) were inserted to the primers and were mixed with the WT primers for quantification. Both general ARMS-PCR and STexS PCRs were identically performed. qPCRs were performed with 790-F49-1, 790-F49-2 and 790-F49-3 for EGFR T790M, and 1 uM of AHP3 as dbOligo for STexS PCR was used.

Amplification refractory mutation system-polymerase chain (ARMS-PCR) is known as a simple and economical method for genotyping SNPs^9^. To demonstrate whether the STexS also contributes in an ARMS-PCR, several primers were designed to target an identical gene region EGFR T790M with (an) additional 1–2 mismatch bases introduced within each primer. As a result, all normal ARMS-PCRs increased the discrimination between wild-type and T790M. When dbOligo was added in each ARMS-PCR, the discrimination was further increased showing ΔΔCt values of 2.39, 3.97 and 3.24 (Fig. 3). Interestingly, the results indicate the effectiveness of STexS for discrimination when adopted in not only AS PCR but also ARMS PCR to detect a single mutation.

### Validation of STexS PCR with various DNA polymerases resulted as an effective way to differentiate SNPs

**Figure 4:**
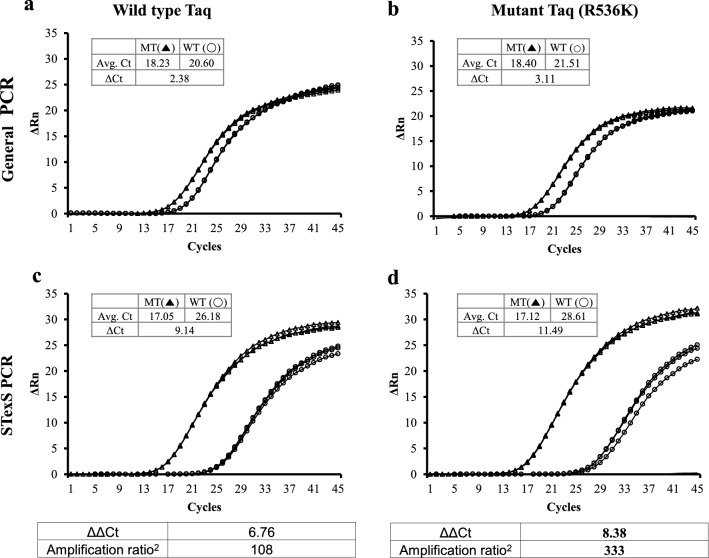
Efficiency of STexS PCR with additional modified DNA polymerase. Each qPCR trial was performed using wild type Taq and mutant Taq (R536K). Each amplification ratio is quantified by ΔΔCt of wild type (**a**, **c**) and mutant (**b**, **d**) Taq. qPCRs were performed with 790-F58-1 for EGFR T790M, and 4 uM of AHP3 as dbOligo for STexS PCR was used. The amplification ratio^2^ was calculated by using the ΔΔCt values of each wild type Taq and mutant Taq to compare between general (**a**, **b**) and STexS (**c**, **d**) PCR.

Recent studies suggest the use of modified DNAPs to enhance the differentiating efficiency of 3’mismatches^10^. To test whether adding modified DNAPs further increase the overall amplification gap between matched and mismatched templates, wild type Taq or mutant Taq (R536K) was added in each standard and STexS PCRs. The mutated Taq polymerase itself increased the discrimination compared to the WT Taq polymerase (Fig. 4a and b). When dbOligo was added to PCR, each ΔΔCt values of wild type Taq and mutant Taq (R536K) were 6.76 and 8.38 (Fig. 4c and d). The result implies that STexS PCRs perform in a synergistic manner when modified DNAP is implemented. Although the performance of the combined STexS and R536K mutated Taq DNAP expressed an impressive outcome, not all SNP targeting genes will be implemented to sufficient discrimination.

**Figure 5:**
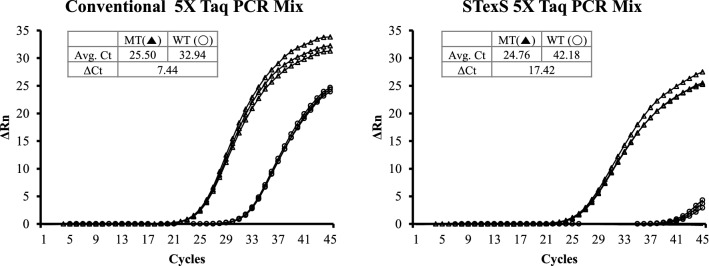
Amplification rates of conventional Taq PCR and STexS implemented Taq PCR. Each qPCR was performed using the conventional 5X Taq PCR mixture. The amplification ratio was calculated using the ΔΔCt value obtained by subtracting each Ct of the WT template and mutated template. qPCRs were performed with 790-F49-2 for EGFR T790M and 4 uM of AHP3 as dbOligo for STexS PCR was used.

STexS PCR was further tested using 5X PCR mixtures that adjusted primer set and dbOligo amount (4 uM) for the high discrimination to evaluate the significance in SNP typing using the considerable amount (1 × 10^5^ copies) of samples. As a result, in a conventional 5X Taq PCR, the difference between matched and mismatched templates was slim. Surprisingly, the STexS PCR expressed drastic contrasts between matched and mismatched, repressing the mismatched template well below the detection value (Fig. 5). Alongside conventionally used PCR mixtures, plasmid copied DNA were tested with commercially used human gDNA to detect possible differences between cell-line made DNA and real samples. Repetitive trials targeting 60 types of SNP resulted in no difference between commercially used human gDNA and plasmid copied DNA (Sup Fig. 7). Overall results show the STexS PCR platform to be a successful method for amplifying mutant DNAs without unwanted duplicated mismatches.

## Discussion

It has been reported that the kinetics of forming the complex (DNAP·P/T) consisting of DNA polymerase (DNAP), primer(P) and template(T) is highly different according to 3’ matched or mismatched^11^. Within the DNA annealing process, 3′ matched complexes (DNAP·P/T_1_) are reported to be 100–1000 times higher of K_cat_/K_m_ than 3′ mismatched complexes (DNAP·P/T_2_). This difference is made by a drastic decrease of K_cat_ (around 10–600 times) and a slight increase of K_m_ (maximum 3 times) caused by 3′ mismatched. According to the kinetics of PCR in the report mentioned above, polymerization is continued without DNA polymerase detach due to high K_cat1_ of 3′ matched complex (DNAP·P/T_1_), but DNA polymerase will repeatedly be detached and attached in the event of discontinuous polymerization due to the low K_cat2_ of 3′ mismatched complex (DNAP·P/T_2_) (Sup Fig. 1c and d). The specifically designed dbOligo focuses on the detachment of DNAP with mismatched templates to further emphasize the amplification ratio of targeted SNPs.

Targeting mutated DNAs within a gene can be beneficial in various circumstances. The importance of SNPs as prognostic or predictive cancer biomarkers are implemented throughout various aspects such as SNP targeting cancer drugs, precision medicine and biomarker derived anticancer therapy^12^. As the development of genomic sequencing continues to improve, more and more mutations within the human gene will be discovered to better predict, diagnose and treat various disorders. Alongside with the increasingly found mutated targets, superior methods and better solvents will be required to detect the elusive SNPs within the abundant normal DNA. To further provide a better platform to detect and easily amplify, we introduce a novel platform regarding the use of oligo-nucleotide DNA. The designed STexS method performed well above the normal AS-PCR to differentiate mutated genes alongside repression of normal genes. The STexS platform also excels in specific PCR methods such as ARMS-PCR and provide a superior method for genotyping SNPs of very small amounts. It is well known that conventional allele-specific PCR lacks in specificity when it comes to SNP detection. The STexS platform shows clear evidence of suppressed amplification in non-specific DNA. This further leads to a decline in false negative rates and will reveal patients which were previously not detected due to undistinguishable amplification rates between mutant and normal DNA. For example, the EGFR gene is well known to be mutated as various forms in non-small cell lung cancer^13^. As it is best to predict such genetic disorders, capturing such mutations in the early stage is essential to lung cancer treatment. The STexS PCR method easily separates the various mutations of EGFR compared to the standard PCR, offering a stable and viable method to detect lung cancer in clinical samples such as blood and lung tissue.

The contents of PCR continue to improve to induce higher activities of polymerases, obtain better amplified copies and reduce unwanted debris such as primer dimers. To better discriminate target SNP from normal DNA, research in polymerase mutation was introduced for the alternative solution. While the mutated DNAP itself was effective at amplifying SNP templates, the synergistic effect of the combined modified DNAP and STexS increased the discrimination ratio^2^ more than 1000 times (ΔΔCt = 9.98) the standard PCR (Fig. 5).

As shown above, the STexS PCR will serve to be an effective method for future SNP detection and give further aid in developing potential biomarkers.

## Materials and methods

### dbOligo design and use

Each dbOligo was designed for efficient forming of double stranded duplex to have at least 10 bases pairing in a single strand. AHP3 is one of the designed dbOligo with the highest performance listed (Sup Table 1). Designed dbOligo were added 1–80 pmol of dOligo per 20 ul of PCR mixtures for the expected 0.05–4 uM of final.

### Allele specific and ARMS PCR

PCR trials were performed using a wild-type and mutated template, forward and reverse primers and dual labeled hydrolysis probes for signal detection (Sup Table 1). Primers for the targeted EGFR gene were designed to match the mutated three variations EGFR T790M, L858R and BRAF V600E alongside mismatching one base pair of each normal genes. All templates for the targets were synthesized artificially and cloned into pUC19 and their copy numbers were measured and calculated with nano drop spectrometry.

Enzymes for each PCR were 2 units of Taq DNA polymerase (GenoTech) to suit the expected 0.05–0.08 uM. PCR buffer were consisted with 10 mM Tris (pH 9.0), 1.5 mM MgCl_2_, 60 mM KCl, 10 mM (NH_4_)_2_SO_4_ mixed to a total of 20 ul. Primers were used 20 pmols and probes were used 10 pmols in 20 ul reactions. Specific cycles were set to the standard ABI 7500 qPCR system, starting from 95℃ to 55℃ for 45 cycles. All trials were tested as a triplicate.

In qPCR, ΔCt = Ct of 3’-mismatched − Ct of 3′-matched, ΔΔCt = ΔCt of STexS − ΔCt of no STexS, amplification ratio^1^ = 2^ΔCt^ and amplification ratio^2^ = 2^ΔΔCt^ were calculated to explain for the discrimination ability.

## Supplementary information

Supplementary Figures.

Supplementary Tables.
