# Supplementary material for: Inclusion of double helix structural oligonucleotide (STexS) results in an enhance of SNP specificity in PCR
Source: Sci Rep. 2021 Sep 27;11:19098. doi: 10.1038/s41598-021-98610-8 (PMC8476546; doi:10.1038/s41598-021-98610-8)
Supplement: Supplementary file 1 — Supplementary Figures. [file 41598_2021_98610_MOESM1_ESM.pptx]

## Slide 1
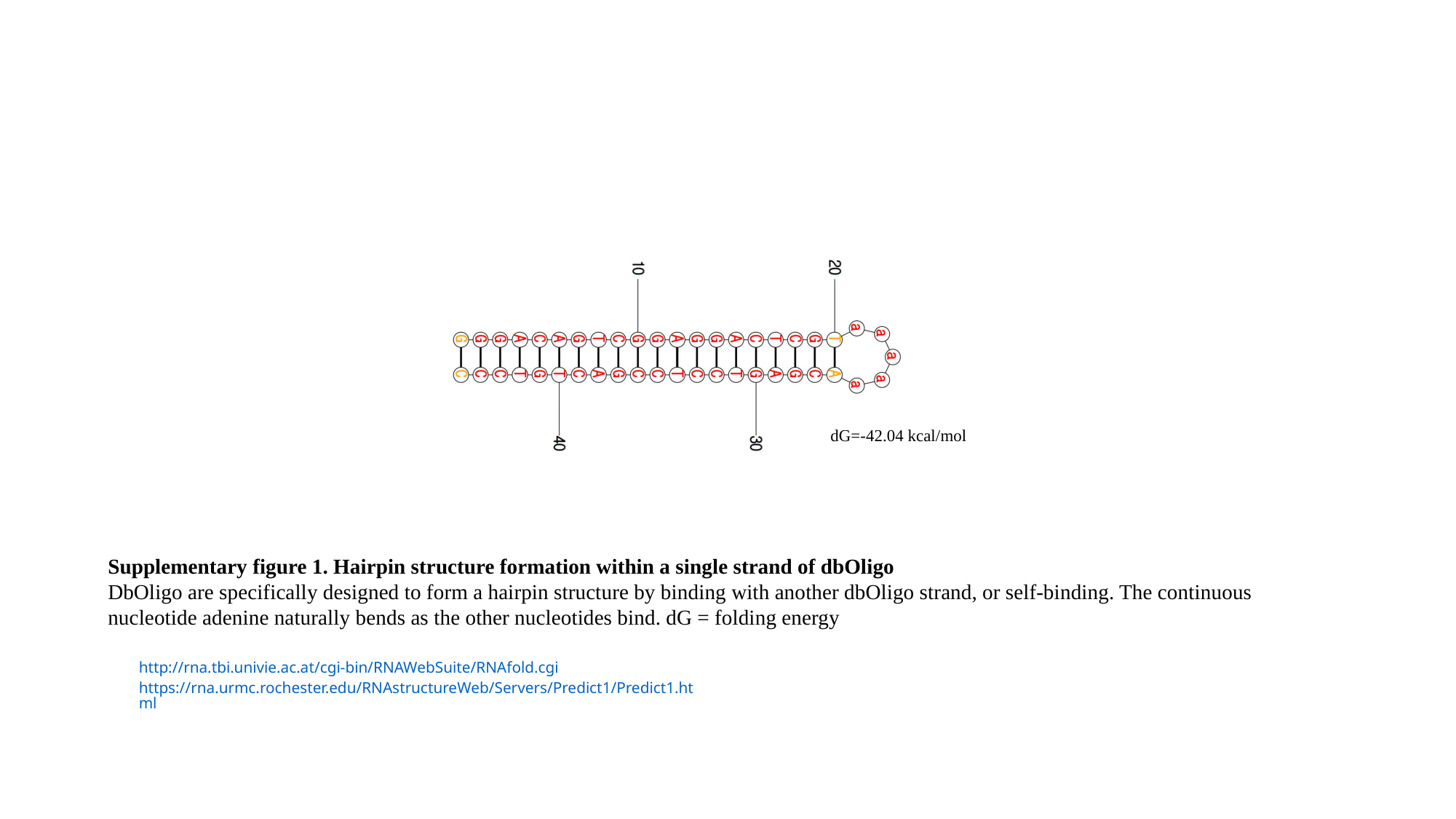

dG=-42.04 kcal/mol
Supplementary figure 1. Hairpin structure formation within a single strand of dbOligo
DbOligo are specifically designed to form a hairpin structure by binding with another dbOligo strand, or self-binding. The continuous nucleotide adenine naturally bends as the other nucleotides bind. dG = folding energy
http://rna.tbi.univie.ac.at/cgi-bin/RNAWebSuite/RNAfold.cgi
https://rna.urmc.rochester.edu/RNAstructureWeb/Servers/Predict1/Predict1.html

## Slide 2
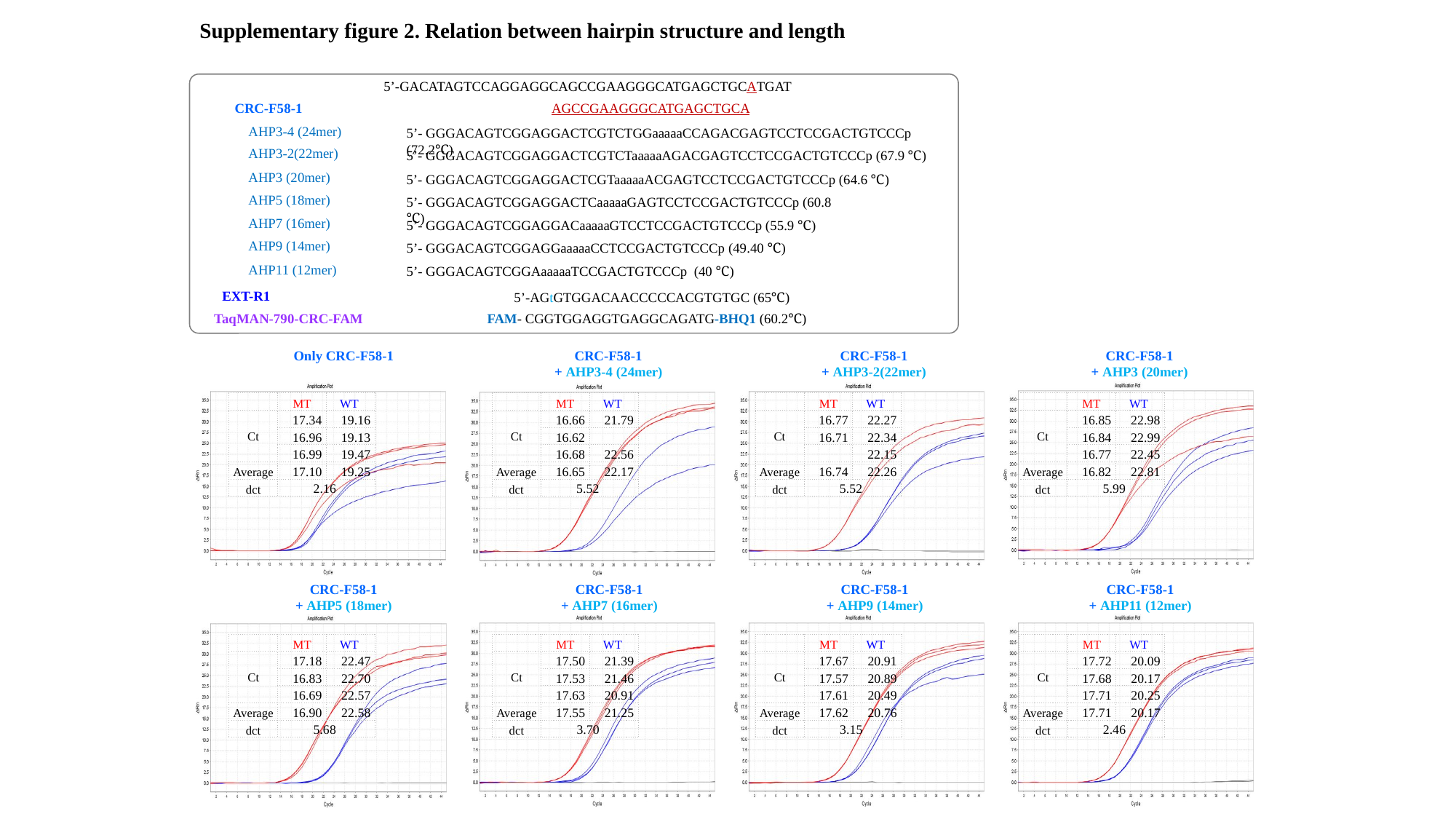

Supplementary figure 2. Relation between hairpin structure and length
5’-GACATAGTCCAGGAGGCAGCCGAAGGGCATGAGCTGCATGAT
AGCCGAAGGGCATGAGCTGCA
CRC-F58-1
AHP3-4 (24mer)
5’- GGGACAGTCGGAGGACTCGTCTGGaaaaaCCAGACGAGTCCTCCGACTGTCCCp (72.2℃)
AHP3-2(22mer)
5’- GGGACAGTCGGAGGACTCGTCTaaaaaAGACGAGTCCTCCGACTGTCCCp (67.9 ℃)
AHP3 (20mer)
5’- GGGACAGTCGGAGGACTCGTaaaaaACGAGTCCTCCGACTGTCCCp (64.6 ℃)
AHP5 (18mer)
5’- GGGACAGTCGGAGGACTCaaaaaGAGTCCTCCGACTGTCCCp (60.8 ℃)
AHP7 (16mer)
5’- GGGACAGTCGGAGGACaaaaaGTCCTCCGACTGTCCCp (55.9 ℃)
AHP9 (14mer)
5’- GGGACAGTCGGAGGaaaaaCCTCCGACTGTCCCp (49.40 ℃)
AHP11 (12mer)
5’- GGGACAGTCGGAaaaaaTCCGACTGTCCCp (40 ℃)
EXT-R1
5’-AGtGTGGACAACCCCCACGTGTGC (65℃)
TaqMAN-790-CRC-FAM
FAM- CGGTGGAGGTGAGGCAGATG-BHQ1 (60.2℃)
Only CRC-F58-1
CRC-F58-1
+ AHP3-4 (24mer)
CRC-F58-1
+ AHP3-2(22mer)
CRC-F58-1
+ AHP3 (20mer)
| | MT | WT |
| --- | --- | --- |
| Ct | 17.34 | 19.16 |
| | 16.96 | 19.13 |
| | 16.99 | 19.47 |
| Average | 17.10 | 19.25 |
| dct | 2.16 | |
| | MT | WT |
| --- | --- | --- |
| Ct | 16.66 | 21.79 |
| | 16.62 | |
| | 16.68 | 22.56 |
| Average | 16.65 | 22.17 |
| dct | 5.52 | |
| | MT | WT |
| --- | --- | --- |
| Ct | 16.77 | 22.27 |
| | 16.71 | 22.34 |
| | | 22.15 |
| Average | 16.74 | 22.26 |
| dct | 5.52 | |
| | MT | WT |
| --- | --- | --- |
| Ct | 16.85 | 22.98 |
| | 16.84 | 22.99 |
| | 16.77 | 22.45 |
| Average | 16.82 | 22.81 |
| dct | 5.99 | |
CRC-F58-1
+ AHP5 (18mer)
CRC-F58-1
+ AHP7 (16mer)
CRC-F58-1
+ AHP9 (14mer)
CRC-F58-1
+ AHP11 (12mer)
| | MT | WT |
| --- | --- | --- |
| Ct | 17.18 | 22.47 |
| | 16.83 | 22.70 |
| | 16.69 | 22.57 |
| Average | 16.90 | 22.58 |
| dct | 5.68 | |
| | MT | WT |
| --- | --- | --- |
| Ct | 17.50 | 21.39 |
| | 17.53 | 21.46 |
| | 17.63 | 20.91 |
| Average | 17.55 | 21.25 |
| dct | 3.70 | |
| | MT | WT |
| --- | --- | --- |
| Ct | 17.67 | 20.91 |
| | 17.57 | 20.89 |
| | 17.61 | 20.49 |
| Average | 17.62 | 20.76 |
| dct | 3.15 | |
| | MT | WT |
| --- | --- | --- |
| Ct | 17.72 | 20.09 |
| | 17.68 | 20.17 |
| | 17.71 | 20.25 |
| Average | 17.71 | 20.17 |
| dct | 2.46 | |

## Slide 3
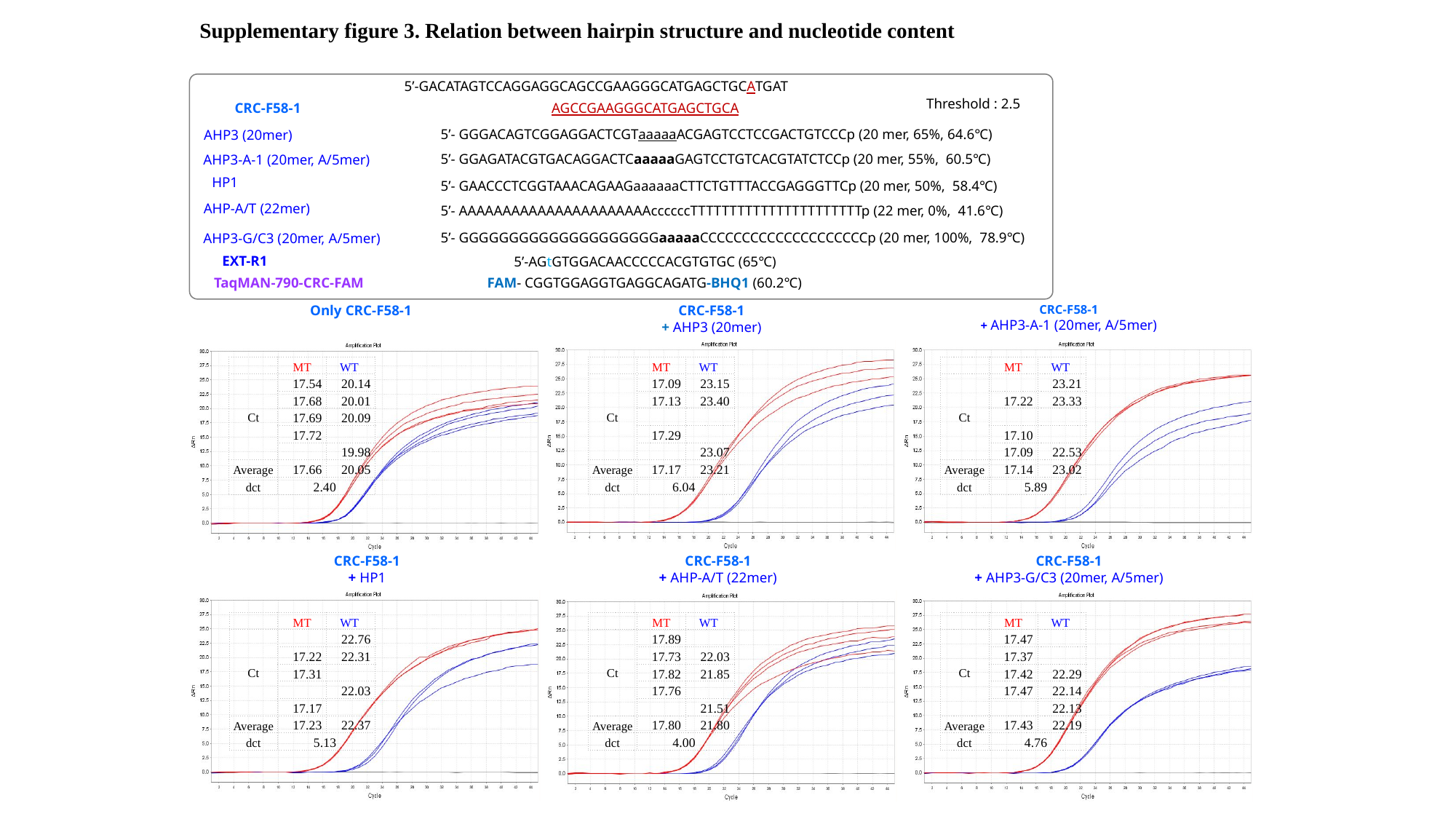

Supplementary figure 3. Relation between hairpin structure and nucleotide content
5’-GACATAGTCCAGGAGGCAGCCGAAGGGCATGAGCTGCATGAT
Threshold : 2.5
AGCCGAAGGGCATGAGCTGCA
CRC-F58-1
5’- GGGACAGTCGGAGGACTCGTaaaaaACGAGTCCTCCGACTGTCCCp (20 mer, 65%, 64.6℃)
AHP3 (20mer)
5’- GGAGATACGTGACAGGACTCaaaaaGAGTCCTGTCACGTATCTCCp (20 mer, 55%, 60.5℃)
AHP3-A-1 (20mer, A/5mer)
HP1
5’- GAACCCTCGGTAAACAGAAGaaaaaaCTTCTGTTTACCGAGGGTTCp (20 mer, 50%, 58.4℃)
AHP-A/T (22mer)
5’- AAAAAAAAAAAAAAAAAAAAAAccccccTTTTTTTTTTTTTTTTTTTTTTp (22 mer, 0%, 41.6℃)
5’- GGGGGGGGGGGGGGGGGGGGaaaaaCCCCCCCCCCCCCCCCCCCCp (20 mer, 100%, 78.9℃)
AHP3-G/C3 (20mer, A/5mer)
EXT-R1
5’-AGtGTGGACAACCCCCACGTGTGC (65℃)
TaqMAN-790-CRC-FAM
FAM- CGGTGGAGGTGAGGCAGATG-BHQ1 (60.2℃)
Only CRC-F58-1
CRC-F58-1
+ AHP3 (20mer)
CRC-F58-1
+ AHP3-A-1 (20mer, A/5mer)
| | MT | WT |
| --- | --- | --- |
| Ct | 17.54 | 20.14 |
| | 17.68 | 20.01 |
| | 17.69 | 20.09 |
| | 17.72 | |
| | | 19.98 |
| Average | 17.66 | 20.05 |
| dct | 2.40 | |
| | MT | WT |
| --- | --- | --- |
| Ct | 17.09 | 23.15 |
| | 17.13 | 23.40 |
| | | |
| | 17.29 | |
| | | 23.07 |
| Average | 17.17 | 23.21 |
| dct | 6.04 | |
| | MT | WT |
| --- | --- | --- |
| Ct | | 23.21 |
| | 17.22 | 23.33 |
| | | |
| | 17.10 | |
| | 17.09 | 22.53 |
| Average | 17.14 | 23.02 |
| dct | 5.89 | |
CRC-F58-1
+ HP1
CRC-F58-1
+ AHP-A/T (22mer)
CRC-F58-1
+ AHP3-G/C3 (20mer, A/5mer)
| | MT | WT |
| --- | --- | --- |
| Ct | | 22.76 |
| | 17.22 | 22.31 |
| | 17.31 | |
| | | 22.03 |
| | 17.17 | |
| Average | 17.23 | 22.37 |
| dct | 5.13 | |
| | MT | WT |
| --- | --- | --- |
| Ct | 17.89 | |
| | 17.73 | 22.03 |
| | 17.82 | 21.85 |
| | 17.76 | |
| | | 21.51 |
| Average | 17.80 | 21.80 |
| dct | 4.00 | |
| | MT | WT |
| --- | --- | --- |
| Ct | 17.47 | |
| | 17.37 | |
| | 17.42 | 22.29 |
| | 17.47 | 22.14 |
| | | 22.13 |
| Average | 17.43 | 22.19 |
| dct | 4.76 | |

## Slide 4
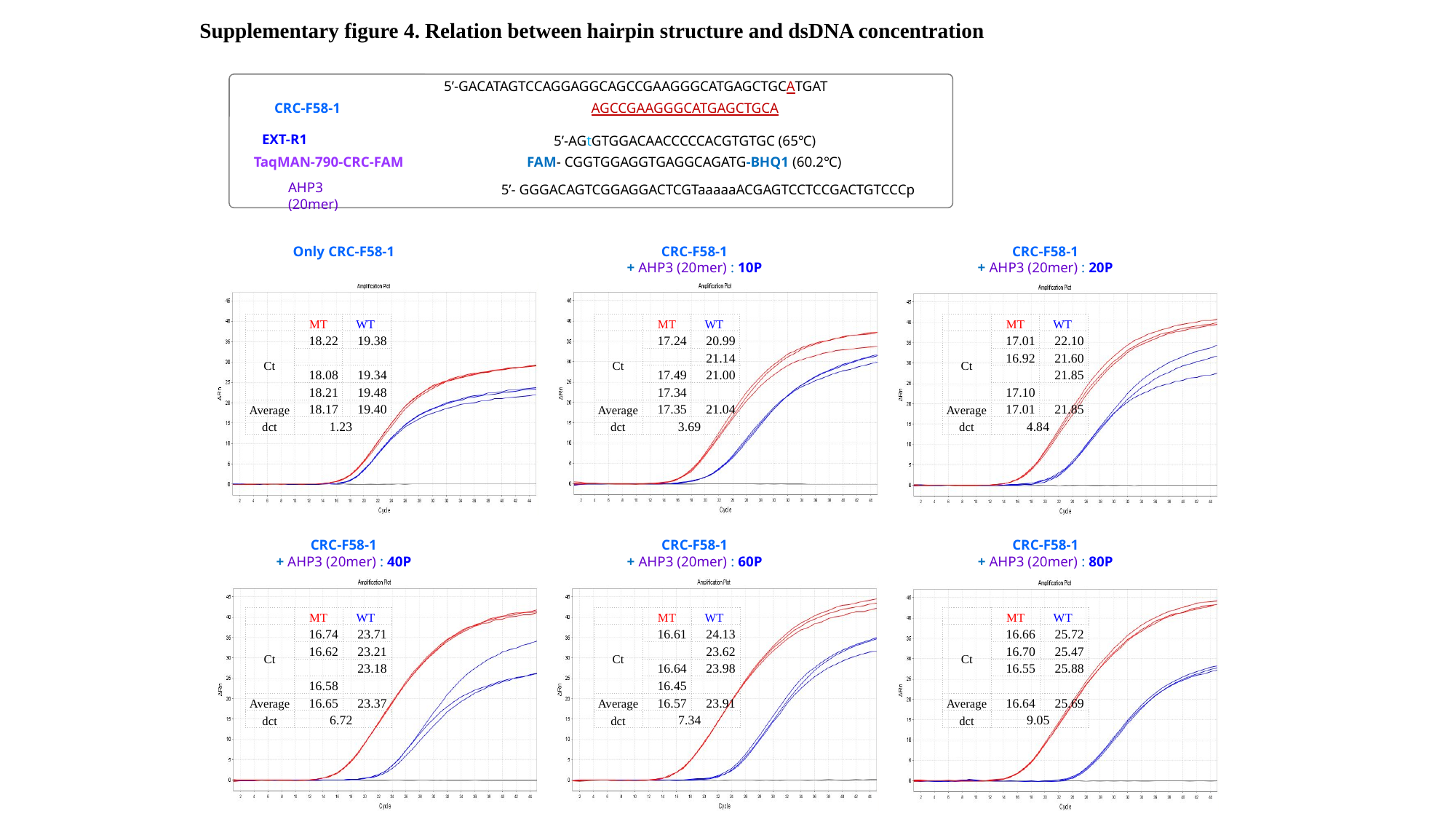

Supplementary figure 4. Relation between hairpin structure and dsDNA concentration
5’-GACATAGTCCAGGAGGCAGCCGAAGGGCATGAGCTGCATGAT
AGCCGAAGGGCATGAGCTGCA
CRC-F58-1
EXT-R1
5’-AGtGTGGACAACCCCCACGTGTGC (65℃)
TaqMAN-790-CRC-FAM
FAM- CGGTGGAGGTGAGGCAGATG-BHQ1 (60.2℃)
AHP3 (20mer)
5’- GGGACAGTCGGAGGACTCGTaaaaaACGAGTCCTCCGACTGTCCCp
Only CRC-F58-1
CRC-F58-1
+ AHP3 (20mer) : 10P
CRC-F58-1
+ AHP3 (20mer) : 20P
| | MT | WT |
| --- | --- | --- |
| Ct | 18.22 | 19.38 |
| | | |
| | 18.08 | 19.34 |
| | 18.21 | 19.48 |
| Average | 18.17 | 19.40 |
| dct | 1.23 | |
| | MT | WT |
| --- | --- | --- |
| Ct | 17.24 | 20.99 |
| | | 21.14 |
| | 17.49 | 21.00 |
| | 17.34 | |
| Average | 17.35 | 21.04 |
| dct | 3.69 | |
| | MT | WT |
| --- | --- | --- |
| Ct | 17.01 | 22.10 |
| | 16.92 | 21.60 |
| | | 21.85 |
| | 17.10 | |
| Average | 17.01 | 21.85 |
| dct | 4.84 | |
CRC-F58-1
+ AHP3 (20mer) : 40P
CRC-F58-1
+ AHP3 (20mer) : 60P
CRC-F58-1
+ AHP3 (20mer) : 80P
| | MT | WT |
| --- | --- | --- |
| Ct | 16.74 | 23.71 |
| | 16.62 | 23.21 |
| | | 23.18 |
| | 16.58 | |
| Average | 16.65 | 23.37 |
| dct | 6.72 | |
| | MT | WT |
| --- | --- | --- |
| Ct | 16.61 | 24.13 |
| | | 23.62 |
| | 16.64 | 23.98 |
| | 16.45 | |
| Average | 16.57 | 23.91 |
| dct | 7.34 | |
| | MT | WT |
| --- | --- | --- |
| Ct | 16.66 | 25.72 |
| | 16.70 | 25.47 |
| | 16.55 | 25.88 |
| | | |
| Average | 16.64 | 25.69 |
| dct | 9.05 | |

## Slide 5
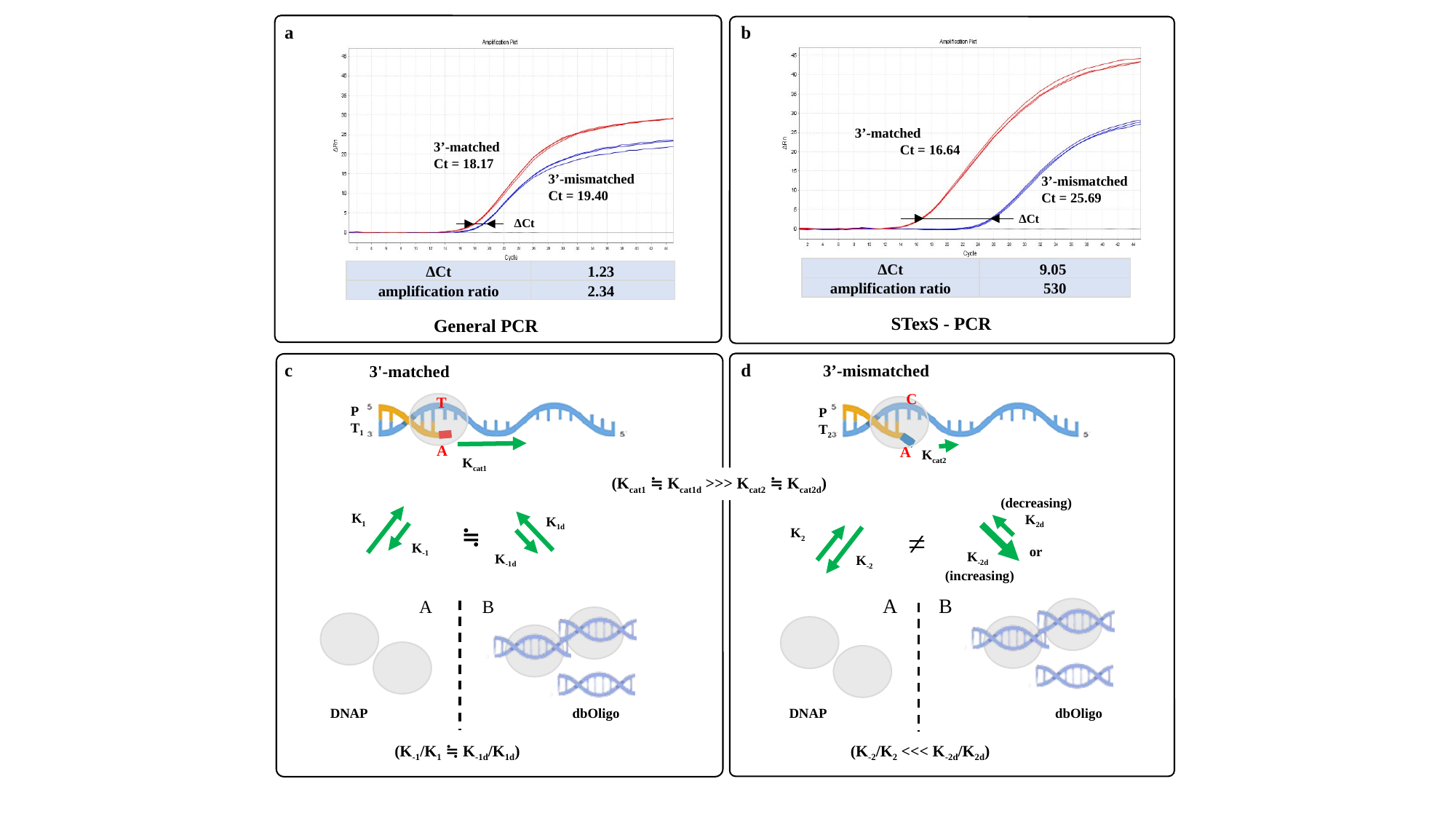

a
b
3’-matched
Ct = 16.64
3’-matched
Ct = 18.17
3’-mismatched
Ct = 19.40
3’-mismatched
Ct = 25.69
ΔCt
ΔCt
| ΔCt | 9.05 |
| --- | --- |
| amplification ratio | 530 |
| ΔCt | 1.23 |
| --- | --- |
| amplification ratio | 2.34 |
STexS - PCR
General PCR
c
d
3’-mismatched
3'-matched
C
T
P
T1
P
T2
A
A
Kcat2
Kcat1
(Kcat1 ≒ Kcat1d >>> Kcat2 ≒ Kcat2d)
(decreasing)
K2d
K1
K1d
 ≒
≠
K2
K-1
or
K-2d
(increasing)
K-1d
K-2
A
B
A
B
DNAP
dbOligo
DNAP
dbOligo
(K-1/K1 ≒ K-1d/K1d)
(K-2/K2 <<< K-2d/K2d)

## Slide 6
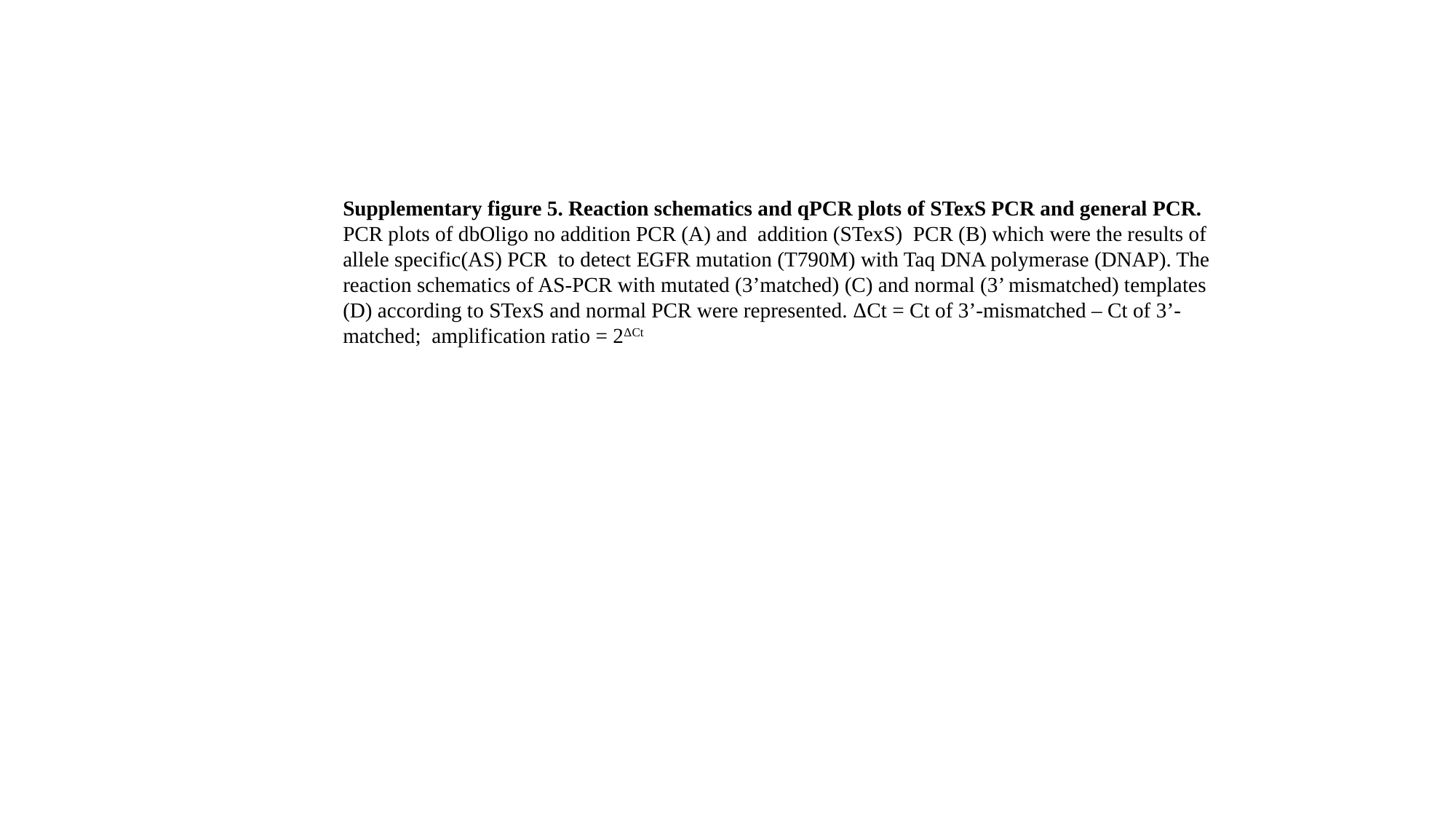

Supplementary figure 5. Reaction schematics and qPCR plots of STexS PCR and general PCR.
PCR plots of dbOligo no addition PCR (A) and addition (STexS) PCR (B) which were the results of allele specific(AS) PCR to detect EGFR mutation (T790M) with Taq DNA polymerase (DNAP). The reaction schematics of AS-PCR with mutated (3’matched) (C) and normal (3’ mismatched) templates (D) according to STexS and normal PCR were represented. ΔCt = Ct of 3’-mismatched – Ct of 3’-matched; amplification ratio = 2ΔCt

## Slide 7
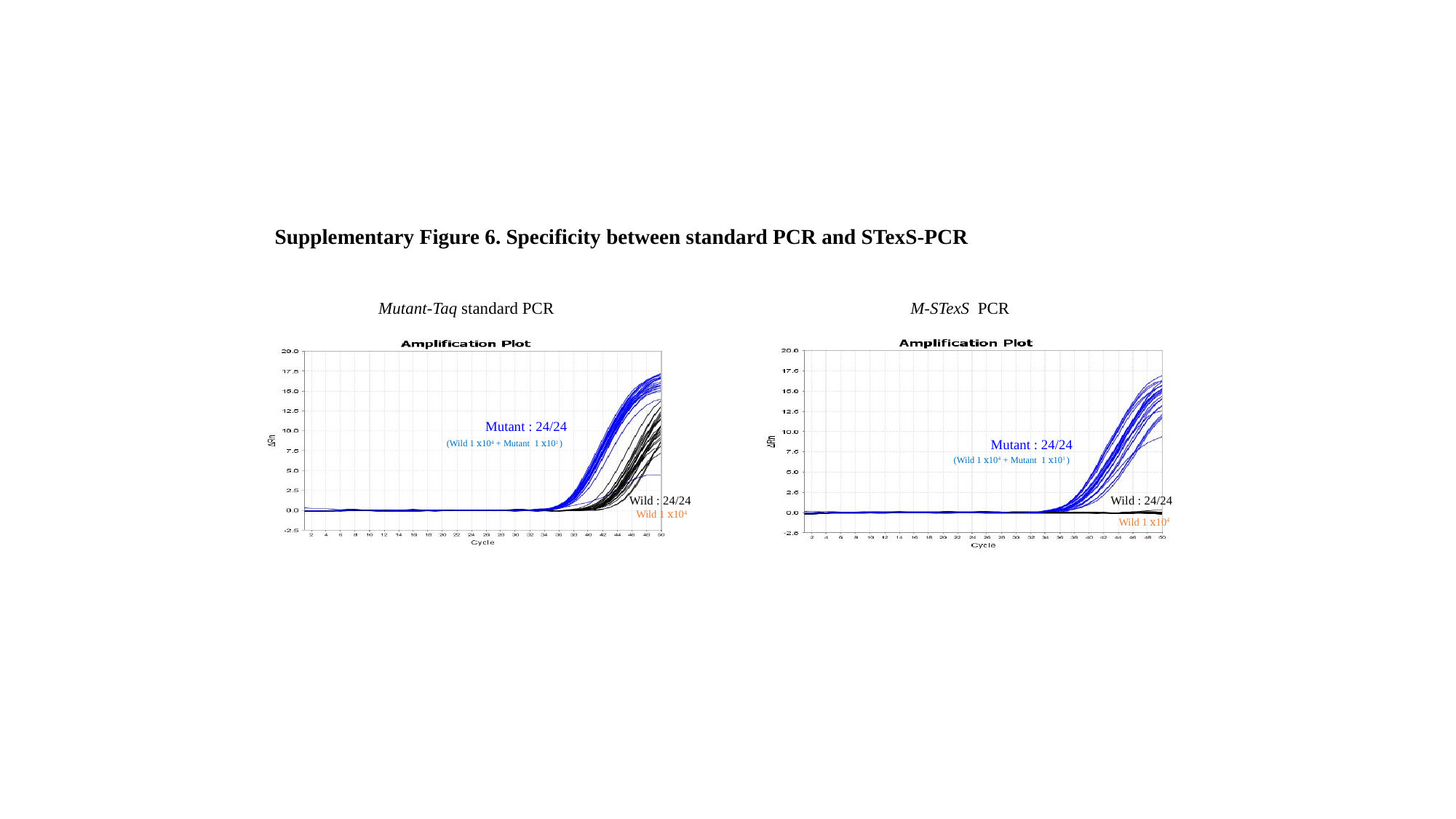

Supplementary Figure 6. Specificity between standard PCR and STexS-PCR
Mutant-Taq standard PCR
M-STexS PCR
Mutant : 24/24
Mutant : 24/24
(Wild 1 ⅹ104 + Mutant 1 ⅹ101 )
(Wild 1 ⅹ104 + Mutant 1 ⅹ101 )
Wild : 24/24
Wild : 24/24
Wild 1 ⅹ104
Wild 1 ⅹ104

## Slide 8
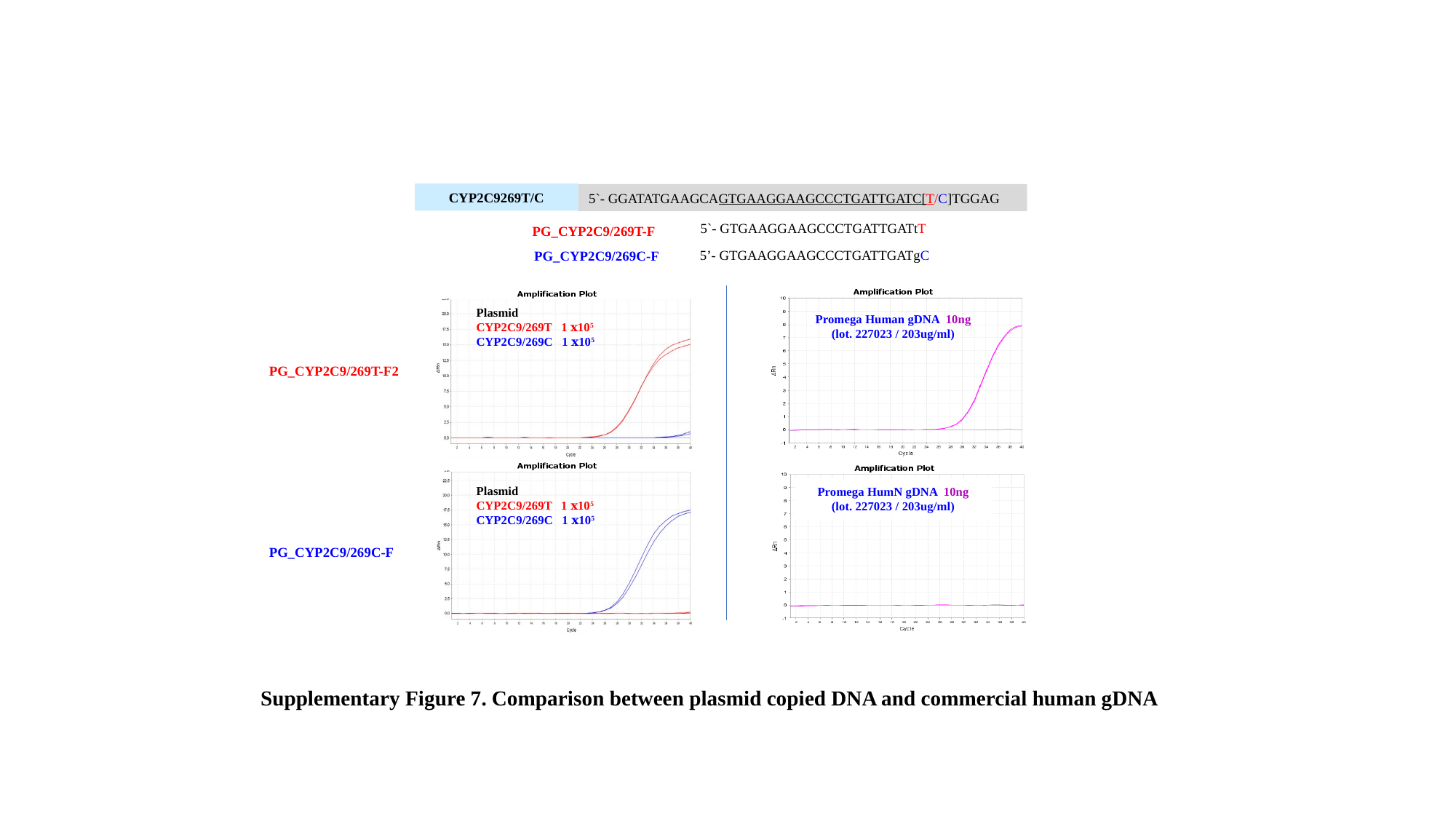

CYP2C9269T/C
5`- GGATATGAAGCAGTGAAGGAAGCCCTGATTGATC[T/C]TGGAG
5`- GTGAAGGAAGCCCTGATTGATtT
PG_CYP2C9/269T-F
5’- GTGAAGGAAGCCCTGATTGATgC
PG_CYP2C9/269C-F
Plasmid
CYP2C9/269T 1 ⅹ105
CYP2C9/269C 1 ⅹ105
Promega Human gDNA 10ng
(lot. 227023 / 203ug/ml)
PG_CYP2C9/269T-F2
Plasmid
CYP2C9/269T 1 ⅹ105
CYP2C9/269C 1 ⅹ105
Promega HumN gDNA 10ng
(lot. 227023 / 203ug/ml)
PG_CYP2C9/269C-F
Supplementary Figure 7. Comparison between plasmid copied DNA and commercial human gDNA
