# Supplementary material for: Inclusion of double helix structural oligonucleotide (STexS) results in an enhance of SNP specificity in PCR
Source: Sci Rep. 2021 Sep 27;11:19098. doi: 10.1038/s41598-021-98610-8 (PMC8476546; doi:10.1038/s41598-021-98610-8)
Supplement: Supplementary file 2 — Supplementary Tables. [file 41598_2021_98610_MOESM2_ESM.docx]

Supplementary Table 1. Used primers, probes and dbOligo

| **Targets /purpose** | **Oligo name** | **Sequence (5'->3')** | **Remark** |
| --- | --- | --- | --- |
| EGFR c.2369 C>T (p.T790M) | 790-F58-1 | AGCCGAAGGGCATGAGCTGC**A** | F-primer |
|  | 790-F49-1 | AGCCGAAGGGCATGAGCTaC**A** | F-primer |
|  | 790-F49-2 | AGCCGAAGGGCATGAGCTGt**A** | F-primer |
|  | 790-F49-3 | AGCCGAAGGGCATGAGCatC**A** | F-primer |
|  | 790-R1 | AGTGTGGACAACCCCCACGTGTGC | R-primer |
|  | 790-FAM | FAM-CGGTGGAGGTGAGGCAGATG-BHQ1 | Probe |
| EGFR c.2573 T>G (p.L858R) | 858-F25 | GCATGTCAAGATCACAGATTTTGGGC**G** | F-primer |
|  | 858-R1 | CTGGCTGACCTAAAGCCACCTC | R-primer |
|  | 858-FAM | FAM-TACCATGCAGAAGGAGGC-BHQ1 | Probe |
| BRAF c.1799 rc. A>T (p.V600E) | 1799-F11 | GGACCCACTCCATCGAGATTTC**T** | F-primer |
|  | 1799-R4 | CACCTCAGATATATTTCTTCATGAAGAC | R-primer |
|  | 1799-FAM | FAM-TAGACCAAAATCACCTATTTTTACTG -BHQ1 | Probe |
| dbOligo | AHP3 | GGGACAGTCGGAGGACTCGTAAAAA*ACGAGTCCTCCGACTGTCCC*p | dbOligo |
